# Tuning the Gas Separation Performances of Smectic Liquid Crystalline Polymer Membranes by Molecular Engineering

**DOI:** 10.3390/membranes12080805

**Published:** 2022-08-20

**Authors:** Joey Kloos, Menno Houben, Johan Lub, Kitty Nijmeijer, Albert P. H. J. Schenning, Zandrie Borneman

**Affiliations:** 1 Membrane Materials and Processes, Department of Chemical Engineering and Chemistry, Eindhoven University of Technology, P.O. Box 513, 5600 MB Eindhoven, The Netherlands; 2 Stimuli-Responsive Functional Materials and Devices, Department of Chemical Engineering and Chemistry, Eindhoven University of Technology, P.O. Box 513, 5600 MB Eindhoven, The Netherlands

**Keywords:** liquid crystal, polymer membranes, gas separation, layer spacing

## Abstract

The effect of layer spacing and halogenation on the gas separation performances of free-standing smectic LC polymer membranes is being investigated by molecular engineering. LC membranes with various layer spacings and halogenated LCs were fabricated while having a planar aligned smectic morphology. Single permeation and sorption data show a correlation between gas diffusion and layer spacing, which results in increasing gas permeabilities with increasing layer spacing while the ideal gas selectivity of He over CO_2_ or He over N_2_ decreases. The calculated diffusion coefficients show a 6-fold increase when going from membranes with a layer spacing of 31.9 Å to membranes with a layer spacing of 45.2 Å, demonstrating that the layer spacing in smectic LC membranes mainly affects the diffusion of gasses rather than their solubility. A comparison of gas sorption and permeation performances of smectic LC membranes with and without halogenated LCs shows only a limited effect of LC halogenation by a slight increase in both solubility and diffusion coefficients for the membranes with halogenated LCs, resulting in a slightly higher gas permeation and increased ideal gas selectivities towards CO_2_. These results show that layer spacing plays an important role in the gas separation performances of smectic LC polymer membranes.

## 1. Introduction

The vast majority of our energy production is generated by the combustion of fossil fuels (e.g., coal and natural gas), which results in enormous amounts of greenhouse gasses such as CO_2_ and CH_4_ being released into the atmosphere, leading to a rise in the average temperature of the Earth’s atmosphere [1,2]. For a sustainable future, greenhouse gas emissions must be reduced, which makes separations such as CO_2_/CH_4_ and CO_2_/N_2_ crucial and relevant [3,4,5,6]. Polymeric membrane processes are, among other technologies, used for gas separation processes because of their low operating costs, high energy efficiency and easy scalability compared to other separation technologies [2,7,8,9]. However, most polymeric membranes used for gas separation are not ordered and aligned at the mesoscopic level, which affects the gas permeability and selectivity [4,8,10,11,12]. Using materials with self-assembly properties to obtain nanostructured polymer membranes provides additional parameters, such as control over the molecular order and alignment of the membrane building blocks, to tailor the gas separation properties. However, the effect of molecular order on the gas separation performance of such nanostructured materials has been rarely reported.

**Figure 1:**
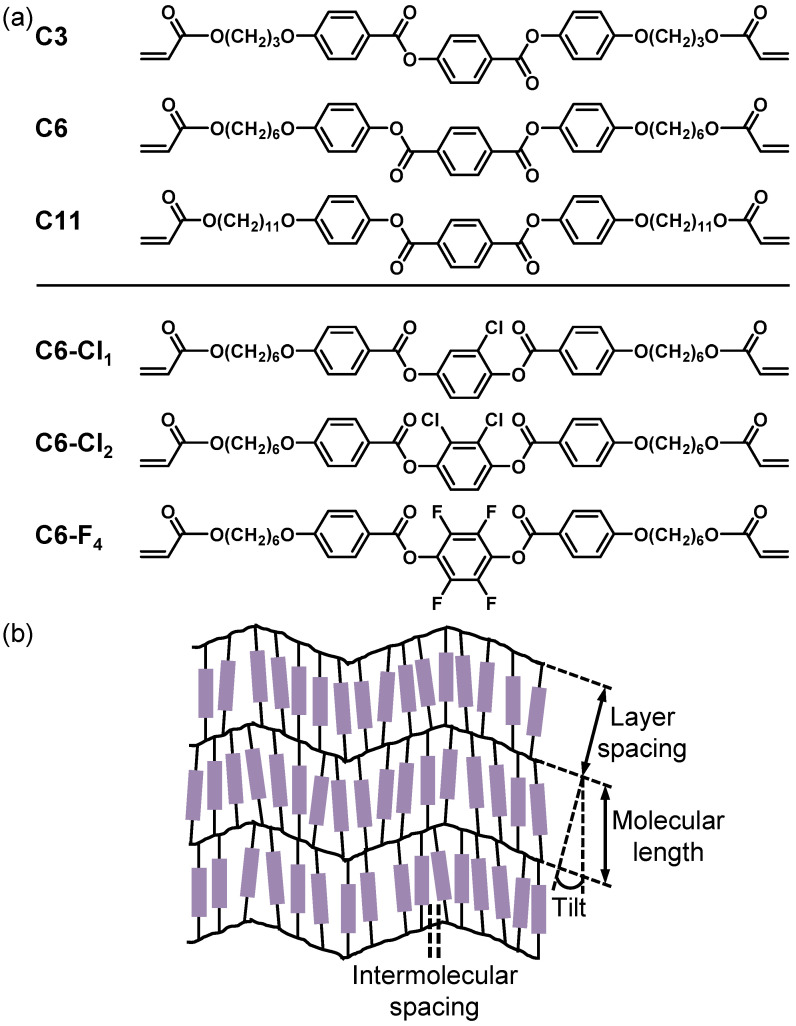
(**a**) Chemical structures of the LCs used in this research. (**b**) Artist impression of a top view of the planar aligned smectic C membranes, which shows the layer spacing, molecular length, intermolecular spacing and tilt angle of the LC molecules. The purple rods represent the aromatic cores of the LCs.

Liquid crystal (LC) molecules can self-assemble into various nanometer structures, which, depending on the positional order of the LC monomers and the fabrication process, can differ in molecular order and orientation. A frequently used fabrication method is to induce self-assembly of reactive LC monomers inside a cell with spacer beads to tune the membrane thickness and alignment layers to control the molecular orientation. Subsequent crosslinking of the LC monomers to fixate the nanostructures is necessary to provide sufficient mechanical strength to obtain robust free-standing LC polymer membranes. So far, LC polymer membranes have mainly been investigated for water separations [13,14,15,16,17,18,19,20,21,22,23,24], but hardly for gas separations [25,26,27]. Studies show the importance of molecular order and orientation in LC polymer membranes, see, for example, our previous work [26]. Here, highly ordered smectic C (lamellar nanostructured) membranes have lower gas permeabilities but selectivity towards He and CO_2_ over N_2_ increases tremendously compared to the membranes without molecular order (see Figure 1b for an artist’s impression of a top view of an LC membrane with a smectic C morphology) [26]. Moreover, the highly ordered smectic C membranes with lamellar structures parallel to the permeation direction (planar alignment) exhibit higher gas permeations but lower selectivities compared to membranes with lamellar structures perpendicular to the permeation direction (homeotropic alignment). However, so far, the role of the dimensions of the nanostructures in the membranes on the gas separation properties has not been reported yet [28]. Moreover, incorporating halogen atoms such as chlorine or fluorine is known to enhance CO_2_ permeability and selectivity by affecting both gas solubility and diffusion [29,30,31,32] and in addition, it also provides a more detailed insight into the gas transport in smectic LC polymer membranes.

Here, we investigate the effects of the layer spacing and halogenation on the gas separation performance of free-standing planar aligned smectic LC polymer membranes for gas separations of He, CO_2_ and N_2_. Various smectic LC membranes are fabricated and characterized by consisting of either LCs with the same functional group but different alkyl spacer length or mixtures of two LCs with the same molecular length but containing different halogenated LCs (see Figure 1a for the LCs used in this research). The effect of layer spacing on the gas separation performances of smectic LC membranes is shown by measuring single gas sorption and permeation of various gasses through membranes with various layer spacings. The effect of halogenation on the gas separation properties of smectic LC membranes is investigated by measuring the single gas sorption and permeation performances of membranes that contain different halogenated LCs.

## 2. Materials and Methods

### 2.1. Chemicals

**Scheme 1:**
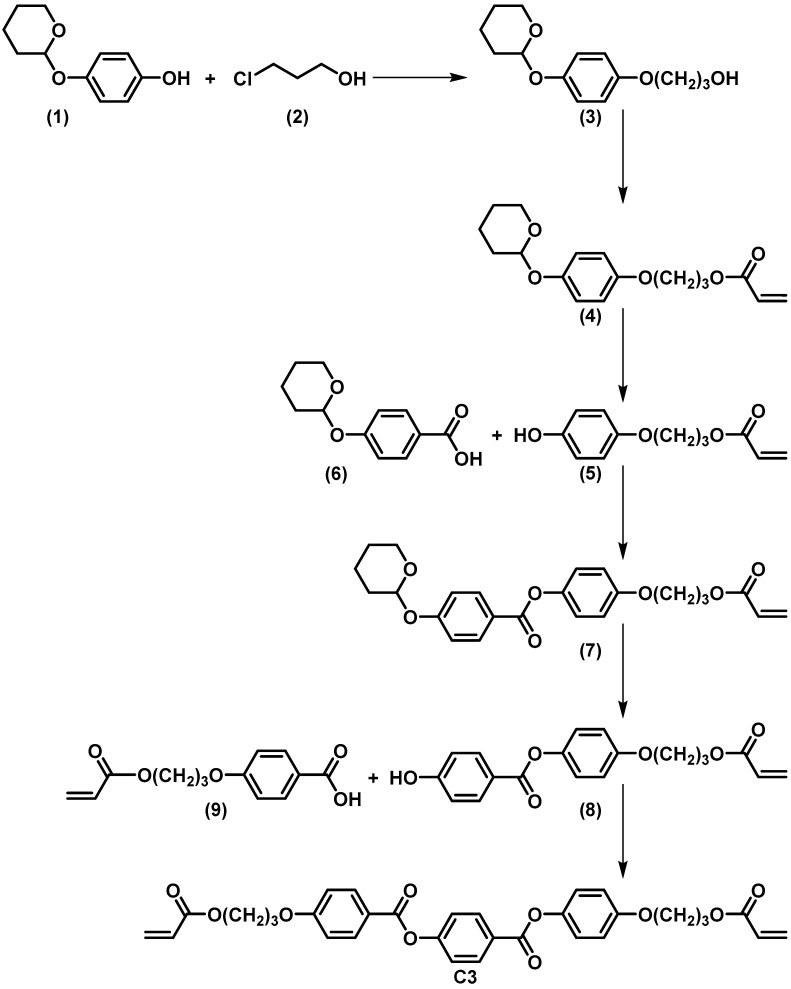
Synthetic route to 4-(3-acryloyloxypropyloxy)phenyl 4-(4-(3- acryloyloxypropyloxy)benzoyloxy) benzoate (**C3**).

**Scheme 2:**
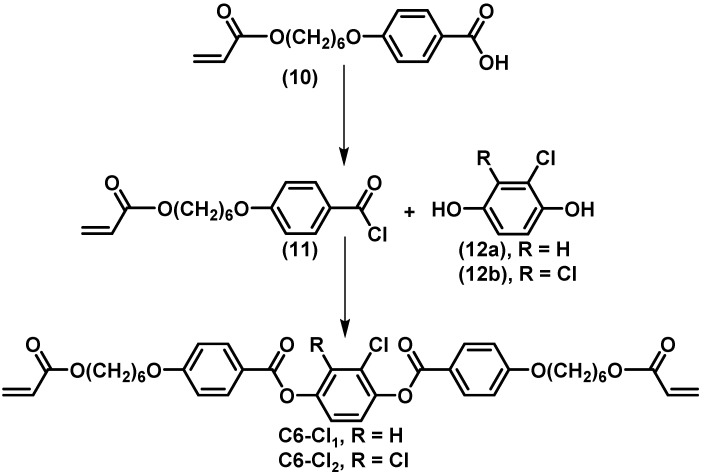
Synthetic routes to 2-chloro-1,4-phenylene bis(4-((6-(acryloyloxy)hexyl)oxy)benzoate) (**C6-Cl_1_**) and 2,3-dichloro-1,4-phenylene bis(4-((6-(acryloyloxy)hexyl)oxy)benzoate) (**C6-Cl_2_**).

The LC diacrylates 1,4-di-(4-(6-acryloyloxyhexyloxy)phenyl)terephthalate (**C6**) [33] and di-4-(11-acryloyloxyundecyloxy)phenyl terephthalate (**C11**) [26] were prepared as described in previous publications. The 2,3,5,6-tetrafluoro-4-(4-(6-acryloyloxyhexyloxy)benzoyloxy)phenyl 4-(6-acryloyloxyhexyloxy)benzoate (**C6-F_4_**) was prepared as described in the literature [34]. The synthesis of 4-(3-acryloyloxypropyloxy)phenyl 4-(4-(3- acryloyloxypropyloxy)benzoyloxy)benzoate (**C3**) is outlined in Scheme 1. (3-(acryloyloxy)propoxy)benzoic acid (**9**) was obtained from Synthon. The 4-((tetrahydro-2H-pyran-2-yl)oxy)phenol (**1**) and 4-((tetrahydro-2H-pyran-2-yl)oxy)benzoic acid (**6**) were both made according to a literature procedure [33]. The syntheses of 2,3-dichloro-1,4-phenylene bis(4-((6-(acryloyloxy)hexyl)oxy)benzoate) (**C6-Cl_2_**) and 2-chloro-1,4-phenylene bis(4-((6-(acryloyloxy)hexyl)oxy)benzoate) (**C6-Cl_1_**) are outlined in Scheme 2. The 4-(6-acryloyloxyhexyloxy)benzoic acid (**10**) was obtained from synthon and 2,3-dichlorohydroquinone (**12a**) was made according to a literature procedure [35].

All other chemicals used for the synthesis of the LC monomers were obtained from Sigma-Aldrich (Zwijndrecht, the Netherlands). The synthetic preparations of the molecules in Scheme 1 and Scheme 2 are described in the supporting information. Irgacure 819 was supplied by Ciba. t-Butyl-hydroquinone was purchased from Merck Life Science (Amsterdam, the Netherlands). For permeation and sorption measurements, the gasses He (5.0 grade), CO_2_ (4.5 grade) and N_2_ (5.0 grade) were obtained from Linde Gas (Schiedam, the Netherlands). All reagents were used as received without further purification.

### 2.2. Membrane Preparation

LC mixtures with 0.5 wt% Irgacure 819 (photoinitiator) and 0.1 wt% t-butyl-hydroquinone (inhibitor) were prepared by dissolving the compounds in a minimum amount of chloroform and subsequently evaporating the solvent (LCs and fabrication conditions are displayed in Table 1, see Figure 1a for chemical structures). Planar aligned smectic C membranes were fabricated by processing the LC mixtures in the smectic phase by capillary suction between two accurately 20 µm spaced glass plates. The glass plates were cleaned before use with isopropanol in an ultrasonic bath for 30 min, dried with N_2_ and treated with UV ozone for 30 min. To obtain planar alignment, the glass plates were functionalized with a rubbed polyimide layer (Optimer AL 1254; JSR Corporation, Toyo, Japan). Glass cells were prepared by gluing two functionalized glass plates together with glue that contained 20 µm glass spacer beads. The filled LC cells were placed inside a temperature-controlled N_2_ box, in which the cells were cooled from the isotropic phase to the smectic phase using a cooling rate of 3 °C/min and subsequently polymerized by exposing the samples for 10 min to an unfiltered spectrum of a collimated EXFO Omnicure S2000 UV lamp (Excelitas Technologies, Germany) with a light intensity of 20 mW/cm^2^ in the range of 320–390 nm. Free-standing membranes were obtained by opening the glass cells in hot water (80 °C).

### 2.3. Characterization

Nuclear magnetic resonance (NMR) spectra were recorded on a 400 MHz Bruker Avance III HD spectrometer in deuterated chloroform with tetramethyl silane (TMS) as the internal standard.

Matrix-assisted laser desorption/ionization time-of-flight mass spectrometry (MALDI-TOF MS) was performed on a Bruker Autoflex Speed MALDI-MS instrument using DCTB (2-[(2E)-3-(4-tert-butylphenyl)-2-methylprop-2-enylidene] malononitril) as matrix.

Attenuated total reflection Fourier transform infrared spectroscopy (ATR FT-IR) spectra were recorded at room temperature on a Varian-Cary 3100 FT-IR spectrometer (Vlissingen, the Netherlands) equipped with a golden gate attenuated total reflectance (ATR) sampling accessory. Scans were taken over a range of 4000−650 cm^−1^ with a spectral resolution of 4 cm^−1^ and 50 scans per spectrum.

Polarizing optical microscopy (POM) was performed using a Leica DM 2700M optical microscope equipped with two polarizers that were operated either crossed or parallel with the sample in between a Linkam hot-stage THMS600 with a Linkam TMS94 controller and a Leica DFC420 C camera.

Differential scanning calorimetry (DSC) measurements were recorded in hermetic T-zero aluminum sample pans using a TA Instruments Q2000 DSC equipped with a cooling accessory. The DSC measurements were performed with three cycles of heating and cooling at a rate of 3 °C/min with an isothermal equilibration of 3 min after each heating or cooling ramp.

Medium- and wide-angle X-ray scattering (MAXS/WAXS) measurements were recorded on a GaneshaLab instrument (Lyngby, Denmark) equipped with a Genix-Cu ultralow divergence source producing X-ray photons of wavelength 1.54 Å and a flux of 108 photons per second. Diffraction patterns were collected on a Pilatus 300 K silicon pixel detector with 487 × 619 pixels of 172 μm^2^.

### 2.4. Gas Sorption

Gas sorption was measured with a Rubotherm series IsoSORP^®^ sorption instrument (Bochum, Germany) to determine the CO_2_ solubility coefficient of all membranes. Before each measurement, the sample was degassed for 5 h by applying a vacuum to the measuring cell followed by a buoyancy measurement with helium to determine the initial sample weight and volume of the sample. Here it was assumed that the solubility of helium is negligible. Gas sorption of CO_2_ was measured at 6 bar and 40 °C (with an equilibrium time of 3 h). The measured sorbed weight was corrected for the buoyancy effect according to Equation (1).

(1) $$m_{\mathrm{corrected}}{=m}_{\mathrm{measured}}{+\rho }_{\mathrm{gas}}\cdot {\;V}_{\mathrm{sample}}\;$$

In Equation (1) m_corrected_ is the corrected weight (g), m_measured_ is the measured weight (g), ρ_gas_ is the density of the measuring gas (g/cm^3^) and V_sample_ is the sample volume (cm^3^). The concentration of CO_2_ was calculated using Equation (2).

(2) $$C_{{\mathrm{CO}}_{2}}=\frac{m_{{\mathrm{CO}}_{2}}\cdot {\;\rho }_{s}}{m_{0}\cdot {\;\rho }_{\mathrm{CO}2}\;\left(\mathrm{STP}\right)}\;$$

In Equation (2) C_CO_2__ is the concentration of CO_2_ in the membrane (cm^3^ (STP)/cm^3^ polymer), m_CO_2__ is the buoyancy corrected mass of CO_2_ in the polymer (g), ρ_s_ is the density of the membrane (g/cm^3^), m_0_ is the initial mass of the sample measured at vacuum (g) and ρ_CO_2__ is the density of CO_2_ gas at standard temperature and pressure (STP) (STP = 273.15 K and 1.013 bar) (g/cm^3^). The CO_2_ solubility coefficient was calculated using Equation (3).

(3) $$S_{{\mathrm{CO}}_{2}\;}=\frac{C_{{\mathrm{CO}}_{2}}}{P}$$

In Equation (3) S_CO_2__ is the gas solubility of CO_2_ (cm^3^ STP/(cm^3^·cmHg)), C_CO_2__ is the concentration of CO_2_ in the membrane (cm^3^ (STP)/cm^3^ polymer) and P is the pressure (cmHg).

### 2.5. Single Gas Membrane Performances

Single gas permeation measurements of He, CO_2_ and N_2_ were performed in a custom-built permeation setup. The flat sheet membranes supported by a Whatman^®^ filter paper (Grade 50 with a pore size of 2.7 µm) to prevent possible pressure-induced punctures, were placed in a stainless-steel cell with a permeation area of 2.1 cm^2^ and the membrane cell was subsequently put in an oven (Convergence Inspector Hephaistos, Enschede, the Netherlands) to control the temperature. Single gas permeabilities were determined from the steady-state pressure increase in time in a calibrated volume at the permeate side of the membrane. The single gas permeabilities were calculated using Equation (4) at 40 °C for two different samples by measuring the permeate pressure increase over time in a calibrated volume with a feed pressure of 6 bar against a vacuum at the permeate side.

(4) $$P_{i}=\frac{\Delta P_{\mathrm{permeate}}\cdot {\;V}_{c}\cdot {\;V}_{m}\cdot L\cdot 10^{10}}{\Delta t\;\cdot R\;\cdot T\;\cdot A\;\cdot \;\Delta P}\;$$

In Equation (4) P_i_ is the permeability of gas species i (Barrer), ΔP_permeate_ is the increase in permeate pressure (Pa) per time interval Δt (s), V_c_ is the calibrated permeate volume (m^3^), V_m_ is the molar volume at STP (cm^3^/mol), L is the membrane thickness (cm), R is the gas constant (J/K·mol), T is the permeate temperature (K), A is the membrane area (cm^2^) and ΔP is the transmembrane pressure (cmHg). The membrane thickness in Equation (4) was determined by taking the average thickness of 7 spots that were measured over the entire membrane area with a micrometer. Before the permeation measurements, the membranes were conditioned overnight (for 12 h) at 6 bar at the feed side with the gas to be measured and vacuum at the permeation side. Subsequently, the permeation of each gas was measured in triplicate on the same membrane. The order of the measured gasses was kept constant for all membranes (He, N_2_ and CO_2_) since CO_2_ could induce swelling of the membrane. The ideal selectivity (α_i/j_) was calculated using the single gas permeabilities as shown in Equation (5), where P_i_ is the permeability of gas species i (Barrer) and P_j_ is the permeability of gas species j (Barrer).

(5) $$\alpha_{i/j}=\frac{P_{i}}{P_{j}}\;$$

The CO_2_ diffusion coefficients of all membranes were calculated by using Equation (6).

(6) $$D=\frac{P}{S}\;$$

In Equation (6), D is the diffusion coefficient (cm^2^/s), P the permeability (Barrer) and S the solubility coefficient (cm^3^ STP/(cm^3^·cmHg)).

## 3. Results and Discussion

### 3.1. Preparation and Characterization of the Liquid Crystalline Molecules and Mixtures

The effect of layer spacing on the gas separation performance of planar aligned smectic LC polymer membranes was studied by varying the length of the alkyl spacers in the LC monomers (**C3**, **C6** and **C11** in Figure 1a). The role of the chemical composition was investigated by incorporating bulky halogen groups such as chlorine or fluorine on the rigid LC core, which are known to enhance CO_2_ permeability and selectivity (**C6-Cl_1_**, **C6-Cl_2_** and **C6-F_4_** in Figure 1a) [29,30,31,32]. The LC monomers **C6, C11** and **C6-F_4_** were synthesized and characterized as described in the literature [26,33,34], while **C3**, **C6-Cl_1_** and **C6-Cl_2_** were synthesized for the first time (see Scheme 1 and Scheme 2 for synthesis schemes). Characterization by ^1^H and ^13^C nuclear magnetic resonance (NMR) and mass spectroscopy (MALDI-TOF MS) confirmed the successful synthesis of all molecules. The phase transitions of all molecules were determined with differential scanning calorimetry (DSC) and polarizing optical microscopy (POM). These results are shown in Table 2 (see Figures S1–S6 for DSC and POM).

DSC and POM (Table 2) show that the **C3**, **C6** and **C11** monomers exhibit nematic and smectic mesophases but at different temperatures. **C11** has the longest flexible alkyl spacer of all synthesized LCs, resulting in the lowest phase transitions. Contrary, **C3** has the shortest alkyl spacer with less flexibility, which leads to the highest phase transitions of all LCs [36]. Only LC mixtures that exhibit a smectic mesophase were used to prepare membranes. Because the pure **C6-Cl_1_**, **C6-Cl_2_** and **C6-F_4_** monomers only exhibit a nematic mesophase, LC mixtures consisting of **C6** with **C6-Cl_1_**, **C6-Cl_2_** and **C6-F_4_** were prepared and characterized with DSC and POM (see Figures S7–S9 for DSC and POM and Table S1 for phase transition values). Compositions containing more than 30 wt% **C6-Cl_1_** or **C6-Cl_2_** and 25 wt% **C6-F_4_** did not exhibit a smectic mesophase and were therefore not used in this study. Hence, LC mixtures consisting of **C6** with respectively 30 wt% **C6-Cl_1_** or **C6-Cl_2_** and 25 wt% **C6-F_4_** were used to prepare membranes to study the effect of halogenation on the gas separation performances of smectic LC membranes.

### 3.2. Preparation and Characterization of Liquid Crystalline Membranes

The LCs were mixed with a photoinitiator to fabricate LC membranes by photopolymerization. Planar-aligned smectic LC membranes were prepared by incorporating the LC mixtures in glass cells with alignment layers to control the orientation of the LCs. After heat treatment, the LC mixtures were photopolymerized in the smectic mesophase to fixate the lamellar morphology. Free-standing LC membranes were obtained by opening the glass cells (see Figure 1b for an artist’s impression of a top view of a planar-aligned smectic C membrane). FT-IR spectra before and after polymerization showed full conversion of the acrylate moieties (Figure S10).

**Figure 2:**
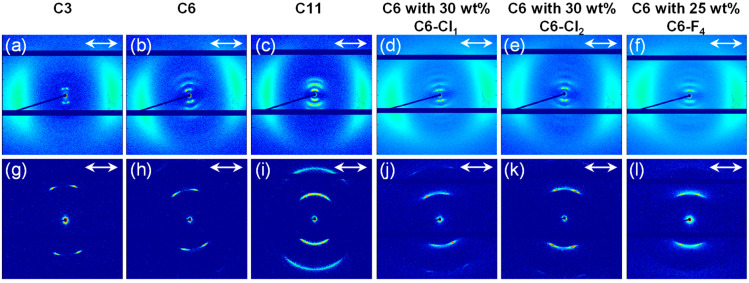
WAXS (top row) and MAXS (bottom row) spectra of LC membranes consisting of LCs with various alkyl spacer lengths and various halogenated LCs. (**a**,**g**) **C3**, (**b**,**h**) **C6**, (**c**,**i**) **C11**, (**d**,**j**) **C6** with 30 wt% **C6-Cl_1_**, (**e**,**k**) **C6** with 30 wt% **C6-Cl_2_**, (**f**,**l**) **C6** with 25 wt% **C6-F_4_**. The single arrow shows the alignment direction.

The alignment and morphology of the membranes were investigated with POM (Figure S11) and showed the planar alignment of all membranes with dark images under parallel conditions and bright images under 45° tilt. Wide-angle X-ray scattering (WAXS) and medium-angle X-ray scattering (MAXS) were measured to further study the morphology and alignment of the membranes (Figure 2).

The 2D WAXS and MAXS spectra in Figure 2 show for all membrane diffraction lobes/spots, indicating that all molecules are aligned. The MAXS spectra of the **C3** (Figure 2g), **C6** (Figure 2h), **C6** with 30 wt% **C6-Cl_1_** (Figure 2j) and **C6** with 30 wt% **C6-Cl_2_** (Figure 2k) membranes show four diffraction spots parallel to the alignment direction, which corresponds to an ordered smectic C morphology. The MAXS spectra of the **C11** membranes (Figure 2i) and the **C6** membranes with 25 wt% **C6-F_4_** (Figure 2l) show diffraction lobes instead of spots, which is characteristic of the morphology between smectic A and smectic C [33]. The more smectic A character of the **C11** membranes compared to the **C6** and **C3** membranes likely arises due to the increased flexibility of the longer alkyl spacer in the **C11** membranes, leading to less stress in the lamellar layers and resulting in a more smectic A morphology. Besides the membrane morphology also the tilt angle, layer spacing and intermolecular spacing were determined from the WAXS and MAXS spectra (Table 3).

Table 3 shows that the tilt angle of the smectic structures is similar for all membranes (varying between 18° and 22°) and is not affected by either extending the alkyl spacer in the LC monomers or by incorporating halogen groups into the LC core. However, the layer spacing, which corresponds to the distance between two layers, is found to be highly dependent on the length of the alkyl spacer in the LC membranes. The determined layer spacing is equal to 31.9 Å for the **C3**, 36.8 Å for the **C6** and 45.2 Å for the **C11** membranes, which is in close agreement with the theoretical extended molecular length of the LC monomers (34.0 Å for **C3**, 40.0 Å for **C6** and 49.9 Å for **C11**). The small difference between the experimentally measured and theoretical layer spacing can be explained by the fact that the flexible alkyl spacers in the molecules are likely folded, leading to slightly lower layer spacing values. Surprisingly, the **C6** membranes that contain halogenated LCs show slightly higher layer spacing values compared to the **C6** membranes without halogenated LCs. The layer spacing further increases with increasing halogen content, leading to the highest layer spacing for the **C6** membranes with 25 wt% **C6-F_4_** (38.1 Å compared to 36.8 Å for the **C6** membranes). Here, the bulky halogen groups can lead to more elongated alkyl chains, which results in slightly higher layer spacing values. The intermolecular spacing that corresponds to the intermolecular stacking of the LC molecules is not affected by the halogen groups and was found to be similar for all membranes (varying between 4.5 and 4.6 Å). The above confirms a planar alignment and smectic morphology for all fabricated LC membranes.

### 3.3. Effect of Layer Spacing on Single Gas Performances

**Figure 3:**
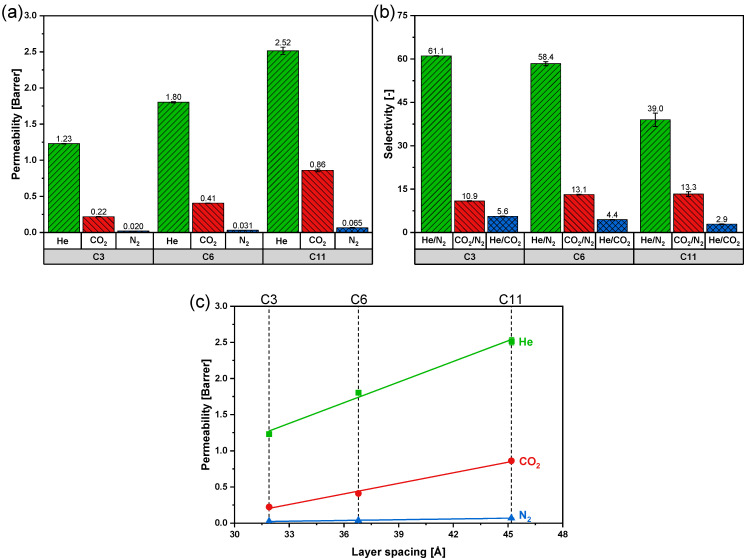
Gas permeation data and ideal gas selectivities of smectic LC membranes with various layer spacings. (**a**) Single gas permeability (He, CO_2_ and N_2_) of **C3**, **C6** and **C11** membranes measured at 40 °C and 6 bar feed pressure. (**b**) Ideal gas selectivities (He/N_2_, CO_2_/N_2_ and He/CO_2_) of **C3**, **C6** and **C11** membranes measured at 40 °C and 6 bar feed pressure. (**c**) Single gas permeability (He, CO_2_ and N_2_) versus the layer spacing of the **C3**, **C6** and **C11** membranes. The small error bars represent the spread of two independently prepared membranes, where each membrane is measured in triplicate.

The effect of the layer spacing on the gas permeation properties of smectic LC membranes was investigated by measuring the single gas permeation of He, CO_2_ and N_2_ at 40 °C in smectic LC membranes with various layer spacings. Permeation data and ideal gas selectivities of He/N_2_, CO_2_/N_2_ and He/CO_2_ are shown in Figure 3 (see Tables S2 and S3 for all permeation and ideal selectivity values). To show the effect of the layer spacing in more detail, the permeation data were also plotted against the layer spacing of the respective membranes (Figure 3c).

Figure 3a clearly shows that the gas permeability increases with increasing alkyl spacer length, resulting in the lowest gas permeabilities for the **C3** membranes and the highest gas permeabilities for the **C11** membranes. Obviously, He has the highest permeability of all membranes, followed by CO_2_ and N_2_. As the gas permeability through dense membranes depends on the kinetic diameter and critical temperature of the gas species (see Table S4 for the kinetic diameter and critical temperature of He, CO_2_ and N_2_) [37]. Helium has the smallest kinetic diameter of all measured gasses, which results in a higher diffusion rate through the membrane and the highest permeability of all gasses. Contrary, N_2_ has the largest kinetic diameter of all measured gasses, leading to a lower diffusion rate through the membrane. Together with a low critical temperature, which affects the condensability of a gas and thereby the solubility in the polymer matrix, this results in the lowest permeability of all measured gasses. CO_2_ has a kinetic diameter between He and N_2_ but has the highest critical temperature of all measured gasses, resulting in a higher solubility and permeability between He and N_2_. Figure 3c shows the relation between layer spacing and gas permeability of smectic LC membranes. The permeability of all gasses decreases with decreasing layer spacing, resulting in the lowest permeabilities for the **C3** membranes with a layer spacing of 31.9 Å, followed by the **C6** and **C11** membranes with layer spacings of, respectively, 36.8 Å and 45.2 Å. This correlation between layer spacing and gas permeability likely arises due to a change in the total free volume within the membrane or/and a change in the size of the free volume elements within the membrane upon a change in layer spacing [30]. A change in the free volume or size of the free volume elements within dense membranes affects the solubility and diffusion of gasses, which can lead to different gas permeabilities. Contrary to the permeability, the ideal gas selectivity of He/N_2_ and He/CO_2_ decreases with increasing layer spacing (Figure 3b), resulting in the highest selectivities for the **C3** membranes (respectively 61.1 for He/N_2_ and 5.6 for He/CO_2_) and the lowest selectivities for the **C11** membranes (respectively 39.0 for He/N_2_ and 2.9 for He/CO_2_). The decrease in selectivity towards He probably originates from the increasing layer spacing of the lamellar structures, which results in larger free volume elements within the membrane. This affects the diffusion of gasses with larger kinetic diameters such as CO_2_ and N_2_ more compared to the smaller He, resulting in a lower selectivity towards He. As the separation of He/N_2_ and He/CO_2_ gas pairs are mainly diffusion-controlled, the selectivities decrease with increasing layer spacing of the lamellar structures. The CO_2_/N_2_ selectivity of the **C6** and **C11** membranes is similar, but the CO_2_/N_2_ selectivity of the **C3** membranes is surprisingly lower compared to the **C6** and **C11** membranes. Similar CO_2_/N_2_ selectivities were expected because the difference in kinetic diameter of CO_2_ and N_2_ (3.30 Å for CO_2_ and 3.64 Å for N_2_) is smaller compared to the difference in kinetic diameter of He and N_2_ (2.60 Å for He and 3.64 Å for N_2_), resulting in comparable diffusion rates of CO_2_ and N_2_ through the membrane. Moreover, the separation of the CO_2_/N_2_ gas pair is mainly sorption-controlled, meaning that these gasses are mainly separated by their differences in gas solubility rather than differences in diffusion. This reduces the influence of the layer spacing on the permeability of CO_2_ and N_2_ and leads to very similar CO_2_/N_2_ selectivities for the **C6** and **C11** membranes.

### 3.4. Effect of Halogenation on Single Gas Performances

**Figure 4:**
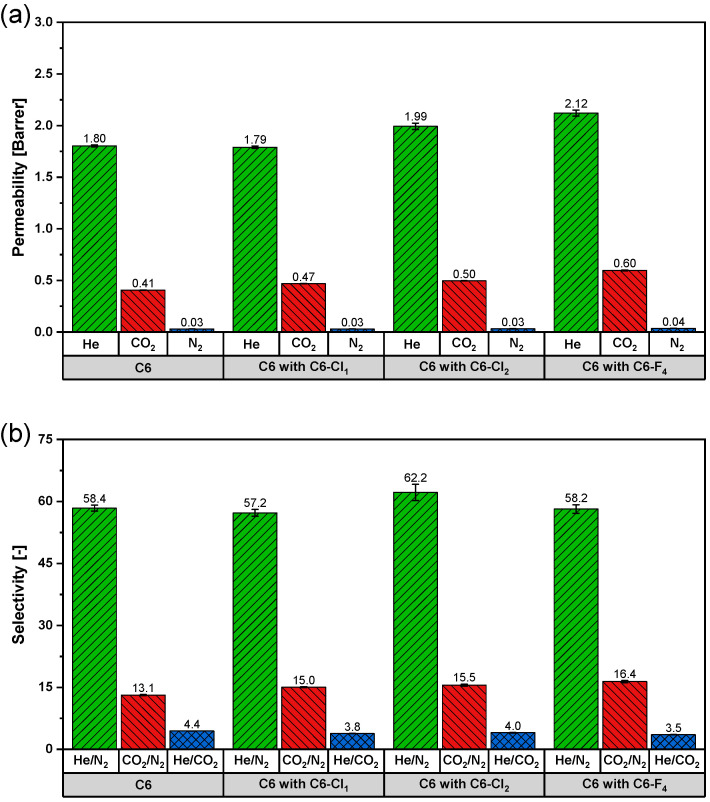
Gas permeation data and ideal gas selectivities of smectic LC membranes with various halogenated LCs. (**a**) Single gas permeability (He, CO_2_ and N_2_) of smectic **C6** membranes with respectively 30 wt% **C6-Cl_1_**, 30 wt% **C6-Cl_2_** and 25 wt% **C6-F_4_** measured at 40 °C and 6 bar feed pressure. (**b**) Ideal gas selectivities (He/N_2_, CO_2_/N_2_ and He/CO_2_) of smectic **C6** membranes with respectively 30 wt% **C6-Cl_1_**, 30 wt% **C6-Cl_2_** and 25 wt% **C6-F_4_** at 40 °C. The small error bars represent the spread of two independently prepared membranes, where each membrane is measured in triplicate.

The effect of halogenation on the gas separation properties of smectic LC membranes was investigated by measuring the single gas permeation of He, CO_2_ and N_2_ at 40 °C for **C6** membranes with various halogenated LCs (respectively 30 wt% **C6-Cl_1_**, 30 wt% **C6-Cl_2_** and 25 wt% **C6-F_4_**). The permeation data and ideal gas selectivities of He/N_2_, CO_2_/N_2_ and He/CO_2_ are shown in Figure 4 (see Tables S5 and S6 for permeation and ideal selectivity values). For comparison, and to show the effect of halogenation in more detail, the permeation and ideal selectivity data of the **C6** membranes without halogenated LCs are also included in Figure 4.

Figure 4a shows that the **C6** membranes with halogenated LCs only exhibit slightly higher permeabilities compared to the **C6** membranes without halogenated LCs. These small differences likely arise from the relatively low halogen content in the halogenated **C6** membranes (30 wt% for the membranes with **C6-Cl_1_** and **C6-Cl_2_** and 25 wt% for the membranes with **C6-F_4_**), which is needed to obtain a smectic morphology. Despite the small permeability differences, the membranes show some subtle variations in permeation behavior. The permeability of all gasses increases with increasing halogen content, resulting in the highest permeabilities for the membranes with 25 wt% **C6-F_4_**. The membranes with 30 wt% **C6-Cl_1_** only show an increase in CO_2_ permeability, while the membranes with **C6-Cl_2_** and **C6-F_4_** show increased permeabilities for all gasses. The increased permeabilities for the membranes with halogenated LCs likely arises due to an increase in the total free volume and/or size of the free volume elements within the membranes with increasing halogen content. The bulky halogen substituents disrupt the chain packing of the molecules, which increases the diffusion coefficient of all gasses, resulting in higher gas permeabilities for the membranes with halogenated LCs compared to the pristine C6 membranes. The CO_2_ permeability of all membranes shows a relatively larger permeability increase compared to He and N_2_. This can be explained two-fold. Firstly, the quadrupole of CO_2_ interacts favorably with the polar halogen groups, which leads to a higher solubility in the polymer matrix and therefore to a higher CO_2_ permeability (Table S3 for quadrupole moments of He, CO_2_ and N_2_). Gasses without these favorable interactions (He) or gasses with only small interactions (N_2_) are less affected by the polarity of the halogen groups, resulting in a lower permeability increase [29,30,31]. Secondly, **C6** membranes that contain halogenated LCs show slightly higher layer spacings compared to the **C6** membranes without halogenated LCs, which also affect the diffusion of gasses through the membranes and lead to an increase in permeability (see Section 3.2 and Section 3.3). Contrary to the membranes with various alkyl spacer lengths, Figure 4b shows that the He/N_2_ selectivity of the **C6** membranes with halogenated LCs is similar for all membranes. Here, the small increase in layer spacing with increasing halogen content does not affect the selectivity towards He. The selectivity towards CO_2_ increases with increasing halogen content due to improved CO_2_-polymer matrix interactions, resulting in higher CO_2_/N_2_ selectivities and lower He/CO_2_ selectivities for the **C6** membranes with halogenated LCs compared to the **C6** membranes. These permeation results show that the used halogen content, which is necessary to obtain smectic LC membranes, only has a limited effect on the gas separation performances (permeability/selectivity) of smectic LC membranes.

### 3.5. CO_2_ Sorption

**Figure 5:**
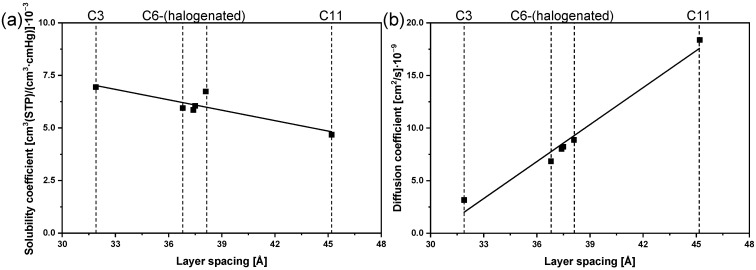
Effect of layer spacing on the CO_2_ (**a**) solubility coefficient (**b**) and diffusion coefficient in smectic LC membranes.

Since the gas permeability of dense membranes is defined as the product of the solubility coefficient and the diffusion coefficient of a certain gas species, CO_2_ sorption was measured to determine the CO_2_ solubility coefficient and further investigate the effect of layer spacing and halogenation on gas solubility and diffusion in smectic LC membranes. The diffusion coefficient was subsequently calculated using Equation (6). Unfortunately, only CO_2_ sorption could be measured because the N_2_ sorption was for all membranes too low to obtain accurate values. The layer spacing, CO_2_ permeabilities, CO_2_ solubility coefficients and the associated calculated diffusion coefficients of all membranes are shown in Table 4. To illustrate the effect of the layer spacing in more detail, the solubility coefficients and the diffusion coefficients were also plotted against the layer spacing of the membranes (Figure 5).

Sorption experiments in Table 4 show that the layer spacing affects both the CO_2_ solubility coefficients and the diffusion coefficients of smectic LC membranes. The solubility coefficients slightly decrease with increasing layer spacing (Figure 5a), resulting in the highest solubility coefficient for the **C3** membranes and the lowest solubility coefficient for the **C11** membranes. This decrease in solubility coefficient with increasing layer spacing can be explained as follows. Varying the alkyl spacer length of the LC monomers not only affects the layer spacing but also the amount of ether/ester groups present in the membrane. The polar ether/ester oxygen groups interact favorably with the quadrupole of CO_2_, which usually leads to higher CO_2_ solubility coefficients for the membranes with more ether/ester groups [38,39,40,41,42]. The ether/ester content of the membranes decreases with increasing alkyl spacer length, resulting in the highest CO_2_ solubility coefficient for the **C3** membranes and lower CO_2_ solubility coefficients for the **C6** and **C11** membranes (**C3** > **C6** > **C11**). Furthermore, the **C6** membranes with halogenated LCs show slightly higher solubility coefficients compared to the **C6** membranes, showing the highest solubility coefficient for the **C6** membranes with 25 wt% **C6-F_4_**. Here, additional favorable interactions between CO_2_ and the polar halogen groups lead to improved CO_2_-polymer matrix interactions, which results in a higher solubility coefficient with increasing halogen content. Contrary to the solubility coefficient, the diffusion coefficient increases tremendously with increasing layer spacing (Figure 5b). The diffusion coefficient of the **C6** membranes is 2 times higher compared to the **C3** membranes, while the **C11** membranes even show a 6-fold increase in the diffusion coefficient compared to the **C3** membranes. This indicates that the increasing gas permeability with increasing layer spacing for the **C3**, **C6** and **C11** membranes can be attributed to an increasing diffusion coefficient. However, the aromatic-aliphatic ratio, which is increasing when going from **C11** to **C3**, might also affect the diffusion coefficient. For the **C6** membranes with halogenated LCs, the slight increase in CO_2_ permeability and selectivity can be attributed to an increase in both solubility and diffusion coefficients. The bulky polar halogen groups in the halogenated **C6** membranes lead to improved CO_2_-polymer matrix interactions and slightly higher layer spacing values, resulting in increased solubility and diffusion coefficients for the **C6** membranes with halogenated LCs compared to the **C6** membranes without halogenated LCs.

The solubility and diffusion coefficients of gasses are highly affected by the available free volume and the size of the free volume elements within the membrane. Usually, both the solubility and diffusion coefficients increase with increasing free volume within a membrane because higher amounts of the free volume provide more sorption sites for gasses. Next to the overall free volume, the diffusion coefficient is also affected by the size of the free volume elements. The activation energy of diffusion is lower for larger free volume elements leading, to a higher diffusion coefficient. The very similar solubility coefficients for all membranes indicate that the overall free volume is equal and is not much affected by either the layer spacing or the bulky polar halogen groups in the smectic structures [43]. It is therefore expected that with increasing layer spacing, not the total free volume within the smectic LC membrane increases, but the free volume elements within the membrane increase in size. This increases the diffusion coefficients for all gasses, leading to higher permeabilities with increasing layer spacing values. However, the diffusion coefficient of gasses with larger kinetic diameters (CO_2_ and N_2_) is more affected by the size of the free volume elements compared to gasses with a smaller kinetic diameter (He), resulting in lower selectivities towards He for the **C11** membranes [44,45].

## 4. Conclusions

The effects of the layer spacing and halogenation on the gas separation performance of free-standing planar aligned smectic LC polymer membranes for gas separations of He, CO_2_ and N_2_ were investigated. All LC membranes consisting of LCs with similar chemical compositions but different alkyl spacer lengths and membranes containing halogenated LCs have a planar alignment and smectic morphology. The tilt angle of the smectic structures was similar for all membranes, but the layer spacing was found to be highly dependent on the length of the alkyl spacer, resulting in smectic LC membranes with various layer spacing values.

Gas sorption of CO_2_ and single gas permeation of He, CO_2_ and N_2_ demonstrated that the permeability increases with increasing layer spacing, while the ideal gas selectivity towards He decreases with increasing layer spacing. It was found that an increasing diffusion coefficient with increasing layer spacing is responsible for the increased permeability, showing that the layer spacing in smectic LC membranes mainly affects the diffusion of gasses rather than their solubility. The effect of incorporating bulky halogens onto the LC cores has been shown to only have a limited effect on the gas permeability and ideal gas selectivity due to the relatively low halogen content in the used membranes, which was needed to maintain a smectic morphology. The CO_2_ permeability of all membranes with halogenated LCs slightly increases with increasing halogen content due to an increase in CO_2_ solubility and diffusion coefficients, resulting in slightly improved selectivities towards CO_2_.

Our results show that especially layer spacing is a crucial parameter that directly controls the diffusion coefficient of gasses in smectic LC membranes and can be used to tune their gas separation performances (permeability/selectivity). Future work should focus on improving the separation performances by reducing the membrane thickness. This research provides insights into the structure-performance relations with single gas measurements, but future research should study the performances under mixed-gas conditions and investigate plasticization and physical aging of the membranes.

## Figures and Tables

**Table 1:** Fabrication conditions for LC membranes with various layer spacings and LC membranes with various halogenated LCs.

Compound/ Mixtures	Isotropic Temperature [°C]	Polymerization Temperature [°C]	Cooling Rate [°C/min]
**C3**	180	100	3
**C6**	155	130	3
**C11**	140	122	3
**C6 with 30 wt% C6-Cl_1_**	140	110	3
**C6 with 30 wt% C6-Cl_2_**	145	115	3
**C6 with 25 wt% C6-F_4_**	140	108	3

**Table 2:** Phase transitions of the synthesized LCs.

Compound	Isotropic [°C]	Nematic [°C]	Smectic [°C]
**C3**	>174	174–113	113–48
**C6**	>146	146–142	142–113
**C11**	>132	132–131	131–116
**C6-Cl_1_**	>110	110–99	-
**C6-Cl_2_**	>139	139–104	-
**C6-F_4_**	>109	109–104	-

**Table 3:** Morphology, tilt angle, layer spacing and intermolecular spacing of the fabricated LC membranes.

Membranes	Morphology	Tilt Angle [°]	Layer Spacing [Å]	Intermolecular Spacing [Å]
**C3**	SmC	21	31.9	4.6
**C6**	SmC	20	36.8	4.6
**C11**	SmA/C	18	45.2	4.6
**C6 with 30 wt% C6-Cl_1_**	SmC	20	37.4	4.5
**C6 with 30 wt% C6-Cl_2_**	SmC	22	37.5	4.6
**C6 with 25 wt% C6-F_4_**	SmA/C	20	38.1	4.5

**Table 4:** The layer spacing, CO_2_ permeabilities, CO_2_ solubility coefficients measured at 40 °C and 6 bar and the associated calculated diffusion coefficients of all membranes.

Membrane	Layer Spacing [Å]	P $\left[\frac{cm^{3}\left(STP\right)\cdot cm}{cm^{2}\cdot s\cdot cmHg}\right]\cdot 10^{-10}\;$	S $\left[\frac{cm^{3}\left(STP\right)}{cm^{3}\cdot cmHg}\right]$$\cdot 10^{-3}$	D $\left[\frac{cm^{2}}{s}\right]\cdot 10^{-9}\;$
**C3**	31.9	0.22	6.94	3.16
**C6**	36.8	0.41	5.95	6.85
**C11**	45.2	0.86	4.68	18.37
**C6 with 30 wt% C6-Cl_1_**	37.4	0.47	5.86	8.01
**C6 with 30 wt% C6-Cl_2_**	37.5	0.50	6.05	8.22
**C6 with 25 wt% C6-F_4_**	38.1	0.60	6.74	8.88

## Supplementary Materials

The following supporting information can be downloaded at: https://www.mdpi.com/article/10.3390/membranes12080805/s1, The synthetic procedures to prepare compounds **C3**, **C6**-**Cl_1_** and **C6**-**Cl_2_** and characterizations of these compounds; Figures S1–S9: DSC and POM images of the compounds and mixtures of **C3**, **C6**, **C11**, **C6**-**Cl_1_**, **C6**-**Cl_2_** and **C6**-**F_4_**; Figure S10: FT-IR of C6 before and after polymerization; Figure S11: POM images of the prepared membranes; Table S1: Phase transitions of the used LC mixtures.; Table S2: Permeabilities of LC membranes with various layer spacings; Table S3: Ideal gas selectivities of LC membranes with various layer spacings; Table S4: Kinetic diameter, critical temperature and quadrupole moment of He, CO_2_ and N_2_; Table S5: Permeabilities of LC membranes with halogenated LCs; Table S6: Ideal gas selectivities of LC membranes with halogenated LCs.
