# Supplementary material for: Tuning the Gas Separation Performances of Smectic Liquid Crystalline Polymer Membranes by Molecular Engineering
Source: Membranes (Basel). 2022 Aug 20;12(8):805. doi: 10.3390/membranes12080805 (PMC9414843; doi:10.3390/membranes12080805)
Supplement: Supplementary file 1 [file membranes-12-00805-s001.zip › membranes-1861620-supplementary.pdf]

membranes

Supplementary Materials

Tuning the Gas Separation Performances of Smectic Liquid Crystalline Polymer Membranes by Molecular Engineering

Joey Kloos [1], Menno Houben [1], Johan Lub [2], Kitty Nijmeijer [1], Albert P. H. J. Schenning [2] and Zandrie Borneman [1,*]

[1] Membrane Materials and Processes, Department of Chemical Engineering and Chemistry, Eindhoven University of Technology, P.O. Box 513, 5600 MB Eindhoven, The Netherlands
[2] Stimuli-responsive Functional Materials and Devices, Department of Chemical Engineering and Chemistry, Eindhoven University of Technology, P.O. Box 513, 5600 MB Eindhoven, The Netherlands
* Correspondence: z.borneman@tue.nl

Citation: Kloos, J.; Houben, M.; Lub, J.; Nijmeijer, K.; Schenning, A.P.H.J.; Borneman, Z. Tuning the Gas Separation Performances of Smectic Liquid Crystalline Polymer Membranes by Molecular Engineering. *Membranes* **2022**, *12*, 805. https://doi.org/10.3390/membranes12080805

Academic Editors: David Alique, David Martinez-Diaz

Received: 27 July 2022
Accepted: 18 August 2022
Published: 22 August 2022

Publisher's Note: MDPI stays neutral with regard to jurisdictional claims in published maps and institutional affiliations.

Copyright: © 2022 by the authors. Licensee MDPI, Basel, Switzerland. This article is an open access article distributed under the terms and conditions of the Creative Commons Attribution (CC BY) license (https://creativecommons.org/licenses/by/4.0/).

Synthesis of 4-(3-acryloyloxypropyloxy)phenyl 4-(4-(3- acryloyloxypropyloxy) benzoyloxy)benzoate (**C3**). See Scheme 1 in the main text.

Synthesis of 3-(4-((tetrahydro-2H-pyran-2-yl)oxy)phenoxy)propan-1-ol **(3)**
5.4 g of sodium methanolate (0.1 mol) was added to a solution of 19.5 g 4-((tetrahydro-2H-pyran-2-yl)oxy)phenol ((1), 0.1 mol) in 80 ml of butanone under nitrogen atmosphere with vigorously stirring. After a clear solution was obtained, 3 g of sodium iodide (0.02 mol) and 8.36 ml of 3-chloropropanol (0.1 mol) were added. The mixture was refluxed for 16 hours and then evaporated under vacuum. 100 ml of diethyl ether and 25 ml of water were added and after separation, the organic layer was extracted twice with 25 ml of an aqueous 10% sodium hydroxide and with 25 ml of brine. After drying over magnesium sulfate and evaporation, an oil was obtained that slowly crystallized. 15.6 g of product **(3)** (62% yield) was obtained as a solid after washing with 150 ml of ligroin and drying over silica in a vacuum desiccator. ^{1}H-NMR (400 MHz, δ in ppm, J in Hz): 6.98 (d, J = 9.0, 2H), 6.82 (d, J = 9.0, 2H), 5.29 (m, 1H), 4.10 (t, J = 6.1, 2H), 3.91 (m, 1H), 3.84 (t, J = 6.1, 2H), 3.60 (m. 1H), 2.05 (p, J = 6.1, 2H), 1.5-2.1 (m, 7H). ^{13}C-NMR (101 MHz, δ in ppm, *: CH or CH_3, #: CH_2): 152.00, 151.20, 118.20*, 115.67*, 97.71*, 66.85#, 62.49#, 61.05#, 32.44#, 30.88#, 25.65#, 19.32.

Synthesis of 3-(4-hydroxyphenoxy)propyl acrylate **(5)**
6.0 ml of acryloyl chloride (74 mmol) was added slowly to a solution of 15.5 g of 3-(4-((tetrahydro-2H-pyran-2-yl)oxy)phenoxy)propan-1-ol (**(3)**, 60 mmol), 9.3 ml of N,N-dimethylaniline (74 mmol) and 20 mg of 4,6-di-tert-butyl-4-methylphenol in 80 ml dichloromethane, stirred in an ice bath under a nitrogen atmosphere. After stirring for 4 hours at room temperature, the solution was subsequently extracted twice with 25 ml of water, 25 ml of an ice-cold aqueous 1N hydrochloric acid solution and 50 ml of brine. The crude intermediate **(4)** was obtained as an oil after drying over magnesium sulfate and evaporation. The crude product was suspended in 70 ml of ethanol that contained 2 g of pyridinium 4-toluensulfonate (0.8 mmol) and refluxed for one hour. The product was precipitated by adding the cooled ethanolic solution to a mixture of 60 g of ice and 120 ml of water with vigorously stirring. 10 g of product **(5)** (74% yield) was obtained as a white solid after washing with 40 ml of water and drying over silica in a vacuum desiccator. ^{1}H-NMR (400 MHz, δ in ppm, J in Hz): 6.70 (m, 4H), 6.43 (dd, *J* = 17.3, 1.5, 1H), 6.14 (dd, *J* = 17.3, 10.4, 1H), 5.85 (dd, *J* = 10.4, 1.5, 1H), 4.84 (s, 1H),), 4.38 (t, *J* = 6.1, 2H), 4.01 (t, *J* = 6.1, 2H), 2.13 (p, J=6.1, 2H). ^{13}C-NMR (101 MHz, δ in ppm, *: CH or CH_3, #: CH_2): 165.91, 153.20, 149.37, 131.31#, 128.75*, 116.44#, 116.04#, 65.41#, 62.12#, 29.12#.

Synthesis of 4-(3-(acryloyloxy)propoxy)phenyl 4-hydroxybenzoate **(8)**

9.3 g of N,N'-dicyclohexylcarbodiimide (44 mmol) was added in small portions to a mixture of 9.8 g of 3-(4-hydroxyphenoxy)propyl acrylate ((5), 44 mmol), 9.8 g 4-((tetrahydro-2H-pyran-2-yl)oxy)benzoic acid (**(6)**, 44 mmol), 0.54 g of N,N-4-dimethylamino-pyridine (4.4 mmol) and 150 ml of dichloromethane stirred in an ice bath under a nitrogen atmosphere. After stirring for 16 hours at room temperature, the solvent was evaporated after filtration over a thin layer of silica. Intermediate **(7)** was obtained as a solid after crystallization from 100 ml of ethanol. The crude intermediate was suspended in 100 ml of ethanol that contained 1.4 g of pyridinium 4-toluensulfonate (0.8 mmol) and refluxed for two hours. The product was precipitated by adding the cooled ethanolic solution to a mixture of 45 g of ice and 90 ml of water with vigorously stirring. 13.3 g of product **(8)** (88% yield) was obtained as a white solid after washing with 40 ml of water and drying over silica in a vacuum desiccator. ^{1}H-NMR (400 MHz, δ in ppm, J in Hz): 8.12 (d, J = 9.1, 2H), 7.11 (d, J = 9.0, 2H), 6.97 (d, J = 8.9, 2H), 6.91 (d, J = 9.1, 2H), 6.43 (dd, *J* = 17.3, 1.5, 1H), 6.13 (dd, *J* = 17.3, 10.4, 1H), 5.90 (s, 1H), 5.86 (dd, *J* = 10.4, 1.5, 1H), 4.38 (t, *J* = 6.1, 2H), 4.08 (t, *J* = 6.1, 2H), 2.15 (p, J=6.1, 2H). ^{13}C-NMR (101 MHz, δ in ppm, *: CH or CH_3, #: CH_2): 164.95, 164,01, 163.95, 156.52, 142.24, 132.94*, 131.31#, 128.75*, 122.33, 122.95*, 115.78#, 115.53#, 65.10#, 61.08#, 29.04#.

Synthesis of 4-(3-acryloyloxypropyloxy)phenyl 4-(4-(3- acryloyloxypropyloxy) benzoyloxy)benzoate (**C3**)

2.1 g of N,N'-dicyclohexylcarbodiimide (10 mmol) was added in small portions to a mixture of 3.4 g 4-(3-(acryloyloxy)propoxy)phenyl 4-hydroxybenzoate (**(8)**, 10 mmol), 2.5 g 4-(3-(acryloyloxy)propoxy)benzoic acid (**(9)**, 10 mmol), 0.12 g of N,N-4-dimethylaminopyridine (1.0 mmol) and 30 ml of dichloromethane stirred in an ice bath under a nitrogen atmosphere. After stirring for 16 hours at room temperature, the solvent was evaporated after filtration over a thin layer of silica. 4.1 g of the final product (**C3**) was obtained as a white solid (71% yield) after crystallization from 50 ml of ethanol that contained 10 mg of 4-methoxyphenol. ^{1}H-NMR (400 MHz, δ in ppm, J in Hz): 8.27 (d, J = 8.7, 2H), 8.16 (d, J = 9.1, 2H), 7.36 (d, J = 8.7, 2H), 7.13 (d, J = 9.0, 2H), 7.00 (d, J = 8.9, 2H), 6.94 (d, J = 9.1, 2H), 6.45 (dd, *J* = 17.3, 1.5, 1H), 6.40 (dd, *J* = 17.3, 1.5, 1H), 6.15 (dd, *J* = 17.3, 10.4, 1H), 6.13 (dd, *J* = 17.3, 10.4, 1H), 5.85 (dd, *J* = 10.4, 1.5, 1H), 5.84 (dd, *J* = 10.4, 1.5, 1H), 4.39 (t, *J* = 6.1, 2H), 4.38 (t, *J* = 6.1, 2H), 4.17 (t, *J* = 6.1, 2H), 4.08 (t, *J* = 6.1, 2H), 2.22 (p, *J* = 6.2, 2H). 2.18 (p, *J* = 6.2, 2H). ^{13}C-NMR (101 MHz, δ in ppm, *: CH or CH_3, #: CH_2): 166.29, 166.25, 164.94, 164.38, 163.47, 156.67, 155.38, 144.59, 132.58*, 131.89*, 131.15*, 131.00*, 128.48#, 128.38#, 127.15, 122.60*, 122.17*, 121.53, 115.29*, 114.54*, 64.89#, 64.86#, 61.45#, 61.27#, 28.79#, 28.65#. HRMS (MALDI-TOF): $[M + Na]^+$ calcd for $C_{32}H_{30}O_{10}Na$: 597.17; found: 597.16.

Synthesis of 2,3-dichloro-1,4-phenylene bis(4-((6-(acryloyloxy)hexyl)oxy)benzoate) (**C6-Cl**$_2$). See Scheme 2 in the main text.

2.6 ml of thionyl chloride (36 mmol) was added dropwise via syringe to a solution of 0.1 ml of N,N-dimethylformamide and 7 g of 4-(6-acryloyloxyhexyloxy)benzoic acid ((10), 24 mmol) in 50 ml diethyl ether cooled in an ice bath and nitrogen was bubbled through the solution. After stirring for one hour at room temperature,10 g of ice and 10 ml of water were added. The organic layer with the crude intermediate acid chloride **(11)** was extracted with 25 ml of brine and dried over $MgSO_4$. The crude product was dissolved in 60 ml of dichloromethane followed by 4.2 ml of triethylamine (30 mmol). 1.8 g of 2,3-dichlorohydroquinone **(12a)**, 10 mmol) was added in portions while the solution was under nitrogen and cooled in an ice bath. After stirring for 48 hours at room temperature, 60 g of ice was added, and the pH was adjusted with concentrated aqueous hydrochloric acid until pH=1. The product was obtained by extracting the organic layer with 60 ml of brine, dried over $MgSO_4$, filtered over a thin layer of silica and the dichloromethane was

subsequently evaporated. 3.4 g of product **C6-Cl$_2$** was obtained as a white solid (47% yield) after crystallization from 35 ml of ethyl acetate that contained 5 mg of 4-methoxyphenol. ^{1}H-NMR (400 MHz, δ in ppm, J in Hz): 8.18 (d, J = 9.0, 4H), 7.28 (s, 2H), 6.99 (d, J = 9.0, 4H), 6.41 (dd, J = 17.3, 1.5, 2H), 6.13 (dd, J = 17.3, 10.4, 2H), 5.82 (dd, J = 10.4, 1.5, 2H), 4.19 (t, J = 6.6, 4H), 4.06 (t, J = 6.4, 4H), 1.85 (p, J = 6.7, 4H), 1.73 (p, J = 7.0, 4H), 1.4-1.6 (m, 8H). ^{13}C-NMR (101 MHz, δ in ppm, *: CH or CH_3, $^{\#}$: CH_2): 166.45, 163.98, 163.81, 146.17, 132.77*, 130.71*, 128.70$^{\#}$, 127.81, 122.07*, 120.64, 114.60*, 68.29$^{\#}$, 64.60$^{\#}$, 29.11$^{\#}$, 28.70$^{\#}$, 25.87$^{\#}$, 25.84$^{\#}$. HRMS (MALDI-TOF): $[M + Na]^+$ calcd for $C_{38}H_{40}Cl_2O_{10}Na$: 749.19; found: 749.17.

Synthesis of 2-chloro-1,4-phenylene bis(4-((6-(acryloyloxy)hexyl)oxy)benzoate) (**C6-Cl$_1$**). See Scheme 2 in the main text.

C6-Cl$_1$ was obtained with a yield of 60% in a similar way as described for the synthesis of 4-di-(4-(6-acryloyloxyhexyloxy)benzoyloxy)-2,3-dichlorobenzene (**C6-Cl$_2$**), but 2,3-dichlorohydroquinone (**12b**) was replaced by 2-chlorohydroquinone (**12a**). ^{1}H-NMR (400 MHz, δ in ppm, J in Hz): 8.18 (d, J = 9.0, 2H), 8.13 (d, J = 9.0, 2H), 7.39 (d, J = 2.7, 1H), 7.32 (d, J = 8.8, 1H), 7.19 (dd, J = 8.8, 2.7, 1H), 6.98 (d, J = 9.0, 2H), 6.97 (d, J = 9.0, 2H), 6.41 (dd, J = 17.3, 1.5, 2H), 6.13 (dd, J = 17.3, 10.4, 2H), 5.82 (dd, J = 10.4, 1.5, 2H), 4.19 (t, J = 6.6, 4H), 4.06 (t, J = 6.5, 4H), 1.85 (p, J = 6.7, 4H), 1.73 (p, J = 7.0, 4H), 1.4-1.6 (m, 8H). ^{13}C-NMR (101 MHz, δ in ppm, *: CH or CH_3, $^{\#}$: CH_2): 166.44, 164.56, 164.06, 163.84, 163.80, 148.81, 145.04, 132.69*, 132.52*, 130.70*, 128.70$^{\#}$, 127.62, 124.35*, 123.92*, 121.31*, 121.15, 120.94, 114.52*, 68.26$^{\#}$, 64.60$^{\#}$, 29.11$^{\#}$, 28.69$^{\#}$, 25.86$^{\#}$, 25.84$^{\#}$. $[M + Na]^+$ calcd for $C_{38}H_{41}ClO_{10}Na$: 715.23; found: 715.22.

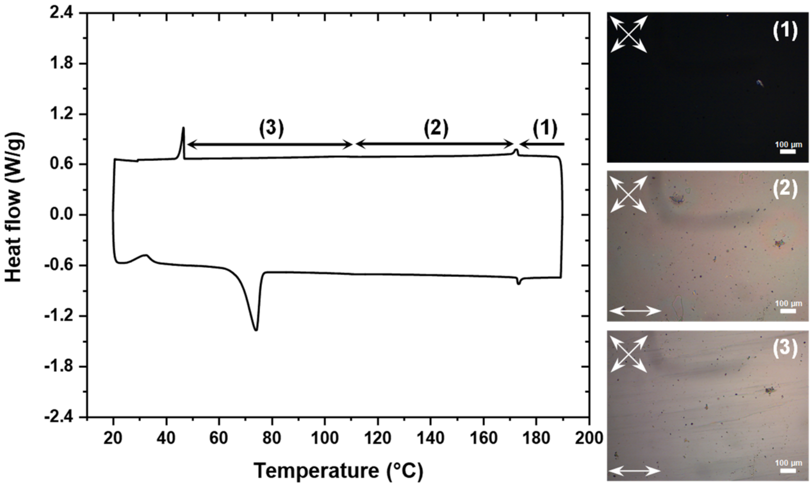

Figure S1. DSC measurement and POM images of **C3**, a photoinitiator and an inhibitor. Measurement was taken at a heating/cooling rate of 3 °C/minute (exothermal down). The POM images correspond to respectively isotropic (1), nematic (2) and smectic (3) phases in the DSC graph. The single arrow represents the alignment direction. The axis of the crossed polarizers are represented by the crossed arrows.

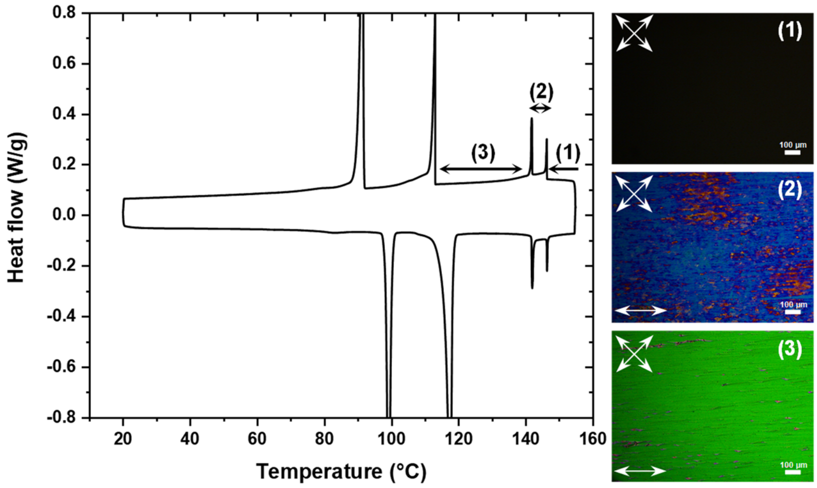

Figure S2. DSC measurement and POM images of **C6**, a photoinitiator and an inhibitor. Measurement was taken at a heating/cooling rate of 3 °C/minute (exothermal down). The POM images correspond to respectively isotropic (1), nematic (2) and smectic (3) phases in the DSC graph. The single arrow represents the alignment direction. The axis of the crossed polarizers are represented by the crossed arrows.

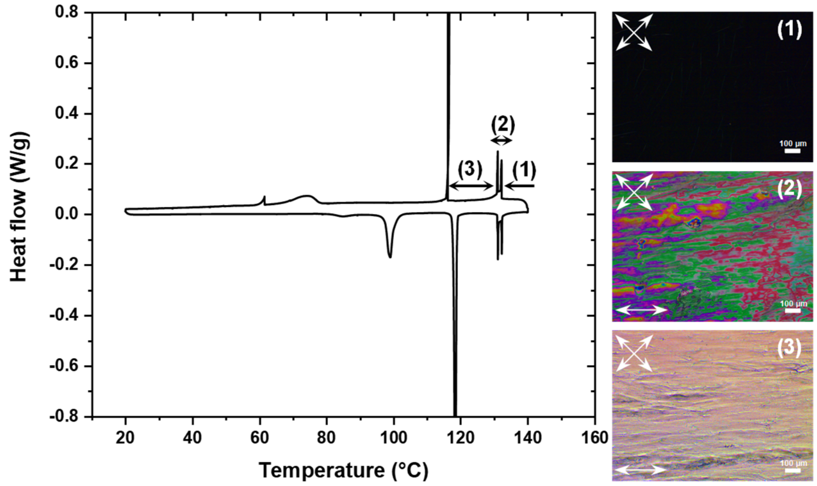

Figure S3. DSC measurement and POM images of **C11**, a photoinitiator and an inhibitor. Measurement was taken at a heating/cooling rate of 3 °C/minute (exothermal down). The POM images correspond to respectively isotropic (1), nematic (2) and smectic (3) phases in the DSC graph. The single arrow represents the alignment direction. The axis of the crossed polarizers are represented by the crossed arrows.

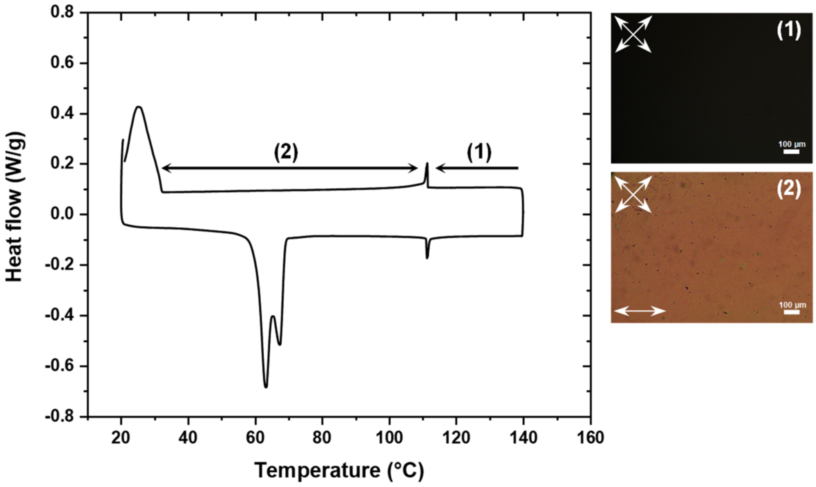

Figure S4. DSC measurement and POM images of **C6-Cl$_1$**, a photoinitiator and an inhibitor. Measurement was taken at a heating/cooling rate of 3 °C/minute (exothermal down). The POM images correspond to respectively isotropic (1) and nematic (2) phases in the DSC graph. The single arrow represents the alignment direction. The axis of the crossed polarizers are represented by the crossed arrows.

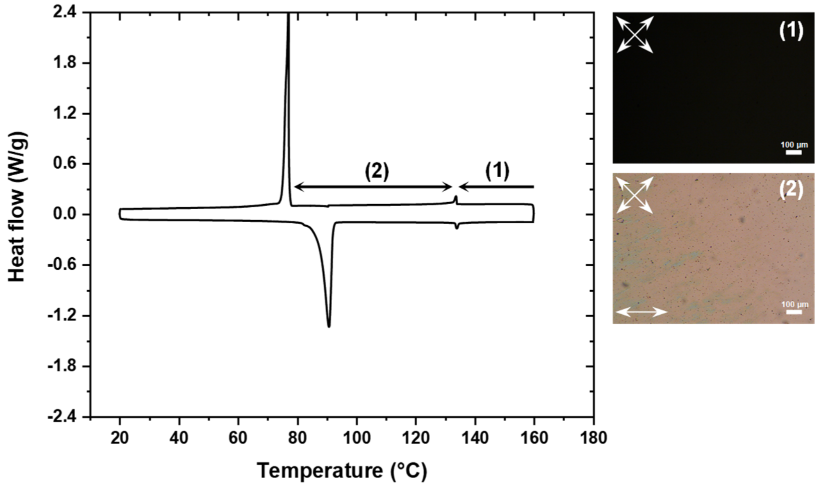

Figure S5. DSC measurement and POM images of **C6-Cl$_2$**, a photoinitiator and an inhibitor. Measurement was taken at a heating/cooling rate of 3 °C/minute (exothermal down). The POM images correspond to respectively isotropic (1) and nematic (2) phases in the DSC graph. The single arrow represents the alignment direction. The axis of the crossed polarizers are represented by the crossed arrows.

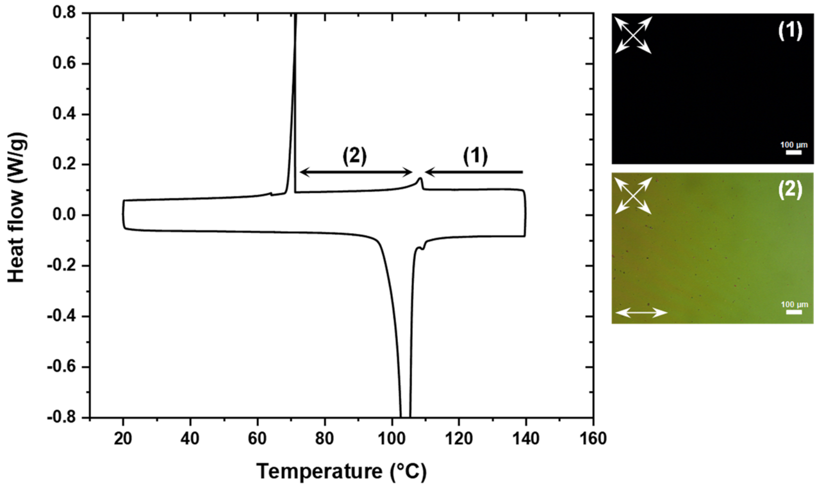

Figure S6. DSC measurement and POM images of **C6-F$_4$**, a photoinitiator and an inhibitor. Measurement was taken at a heating/cooling rate of 3 °C/minute (exothermal down). The POM images correspond to respectively isotropic (1) and nematic (2) phases in the DSC graph. The single arrow represents the alignment direction. The axis of the crossed polarizers are represented by the crossed arrows.

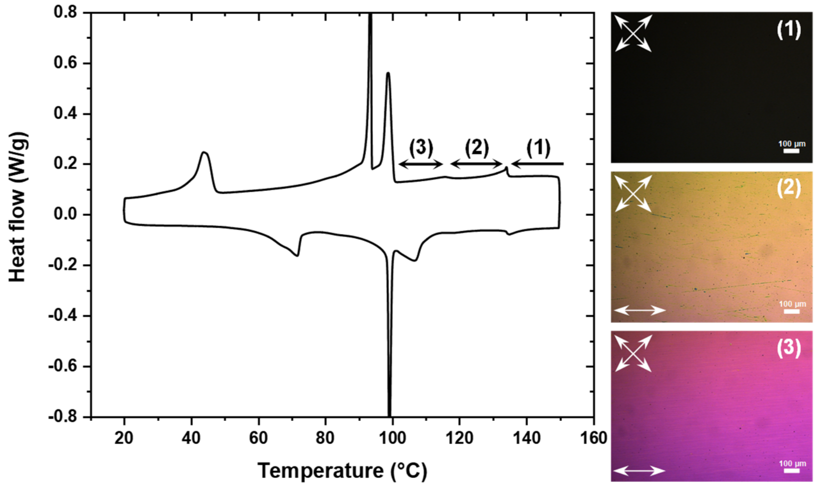

Figure S7. DSC measurement and POM images of a LC mixture consisting of a 7:3 ratio (in wt%) between **C6** and **C6-Cl$_1$**, a photoinitiator and an inhibitor. Measurement was taken at a heating/cooling rate of 3 °C/minute (exothermal down). The POM images correspond to respectively isotropic (1), nematic (2) and smectic (3) phases in the DSC graph. The single arrow represents the alignment direction. The axis of the crossed polarizers are represented by the crossed arrows.

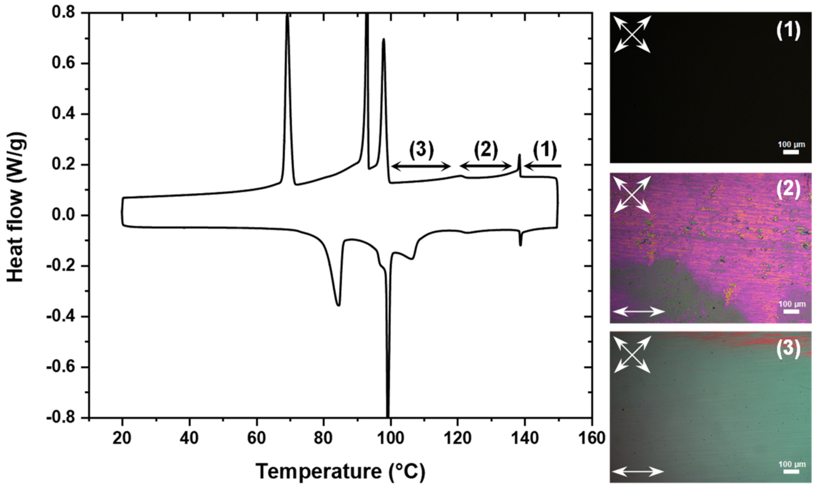

Figure S8. DSC measurement and POM images of a LC consisting of a 7:3 ratio (in wt%) between **C6** and **C6-Cl₂**, a photoinitiator and an inhibitor. Measurement was taken at a heating/cooling rate of 3 °C/minute (exothermal down). The POM images correspond to respectively isotropic (1), nematic (2) and smectic (3) phases in the DSC graph. The single arrow represents the alignment direction. The axis of the crossed polarizers are represented by the crossed arrows.

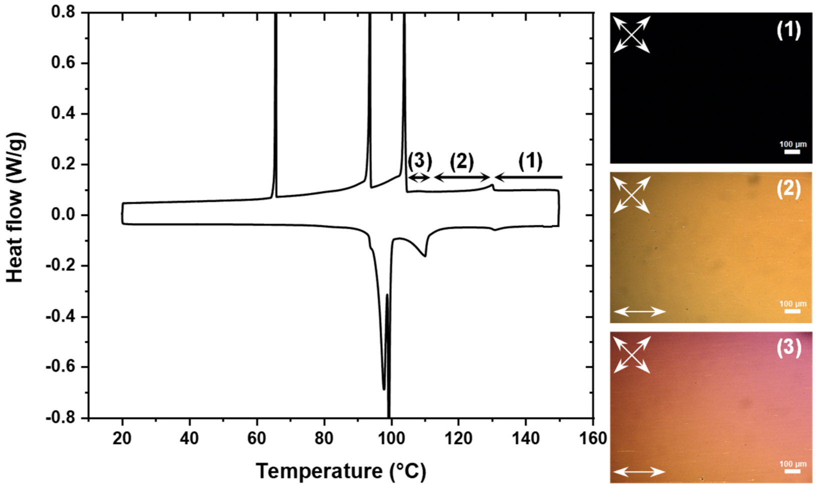

Figure S9. DSC measurement and POM images of a LC consisting of a 3:1 ratio (in wt%) between **C6** and **C6-F₄**, a photoinitiator and an inhibitor. Measurement was taken at a heating/cooling rate of 3 °C/minute (exothermal down). The POM images correspond to respectively isotropic (1), nematic (2) and smectic (3) phases in the DSC graph. The single arrow represents the alignment direction. The axis of the crossed polarizers are represented by the crossed arrows.

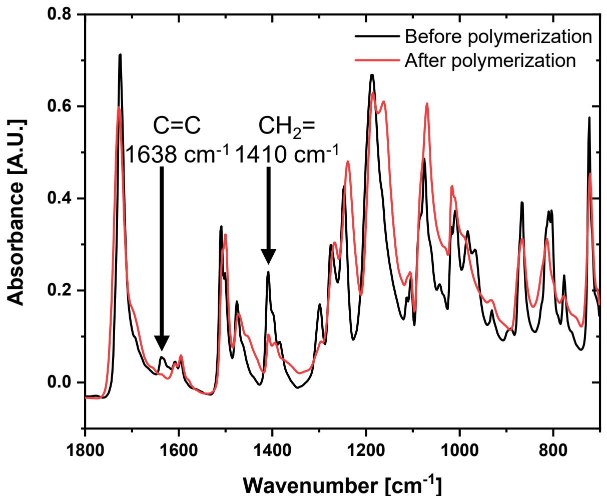

Figure S10. FT-IR analysis of **C6** before and after polymerization. All other LCs show similar graphs before and after polymerization.

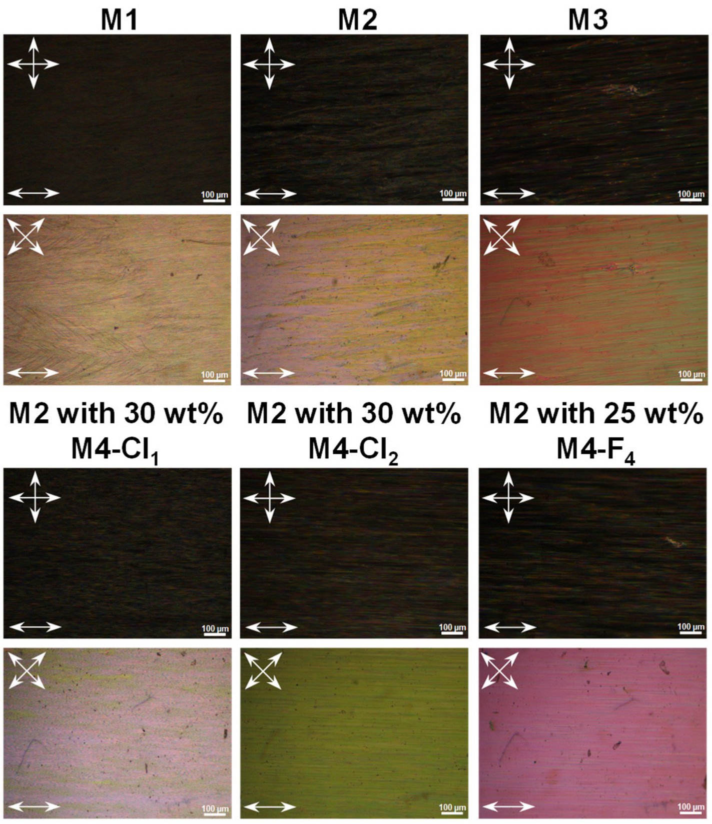

Figure S11. POM images of the polymerized LC membranes. POM images show different birefringence caused by molecular order and orientation. The single arrow represents the alignment direction. The axes of the crossed polarizers are represented by the crossed arrows.

Table S1. Phase transition values of the used LC mixtures.

Compound/ Mixtures	Isotropic [°C]	Nematic [°C]	Smectic C [°C]
**C3**	>174	174-113	113-48
**C6**	>146	146-142	142-113
**C11**	>132	132-131	131-116
**C6 with 30 wt% C6-Cl_1**	>134	134-115	115-101
**C6 with 30 wt% C6-Cl_2**	>139	139-122	122-100
**C6 with 25 wt% C6-F_4**	>131	131-110	110-105

Table S2. Permeabilities (He, CO_2 and N_2) of smectic LC membranes with various layer spacings at 40 °C.

Permeability in Barrer [(cm^3*cm)/(cm^2*s*cmHg)] at 40 °C			
**Gas**	**C3**	**C6**	**C11**
**He**	1.23 ± 0.01	1.80 ± 0.01	2.52 ± 0.05
**CO_2**	0.22 ± 0.01	0.41 ± 0.01	0.86 ± 0.02
**N_2**	0.020 ± 0.001	0.031 ± 0.001	0.065 ± 0.004

Table S3. Ideal gas selectivities of smectic LC membranes with various layer spacings at 40 °C.

Ideal selectivity at 40 °C			
**Gas pair**	**C3**	**C6**	**C11**
**He/N_2**	61.1 ± 0.1	58.4 ± 0.7	39.0 ± 2.3
**CO_2/N_2**	10.9 ± 0.1	13.1 ± 0.1	13.3 ± 0.8
**He/CO_2**	5.6 ± 0.1	4.4 ± 0.1	2.9 ± 0.1

Table S4. The kinetic diameter, critical temperature and quadrupole moment of He, CO_2 and N_2.

Gas species	Kinetic diameter [Å]	Critical temperature [K]	Quadrupole moment [cm^2] ·10^{40}
**He**	2.60	5.19	0.00
**CO_2**	3.30	304.13	-13.71
**N_2**	3.64	126.20	-4.91

Table S5. Permeabilities (He, CO_2 and N_2) of smectic LC membranes with halogenated LCs at 40 °C.

Permeability in Barrer [(cC*cm)/(cm^2*s*cmHg)] at 40 °C				
**Gas**	**C6**	**C6 with 30 wt% C6-Cl_1**	**C6 with 30 wt% C6-Cl_2**	**C6 with 25 wt% C6-F_4**
**He**	1.80 ± 0.01	1.79 ± 0.01	1.99 ± 0.03	2.12 ± 0.03
**CO_2**	0.41 ± 0.01	0.47 ± 0.01	0.50 ± 0.01	0.60 ± 0.01
**N_2**	0.031 ± 0.001	0.031 ± 0.001	0.032 ± 0.001	0.037 ± 0.001

Table S6. Ideal gas selectivities of smectic LC membranes with halogenated LCs at 40 °C.

Ideal selectivity at 40 °C				
**Gas pair**	**C6**	**C6 with 30 wt% C6-Cl_1**	**C6 with 30 wt% C6-Cl_2**	**C6 with 25 wt% C6-F_4**
**He/N_2**	58.4 ± 0.7	57.2 ± 0.8	62.2 ± 2.0	58.2 ± 1.1
**CO_2/N_2**	13.1 ± 0.1	15.0 ± 0.1	15.5 ± 0.3	16.4 ± 0.2
**He/CO_2**	4.4 ± 0.1	3.8 ± 0.1	4.0 ± 0.1	3.5 ± 0.1
